# Morphologic and Molecular Features of Antibody-Mediated Transplant Rejection: Pivotal Role of Molecular Injury as an Independent Predictor of Renal Allograft Functional Decline

**DOI:** 10.3389/ti.2023.12135

**Published:** 2023-12-19

**Authors:** Carsten T. Herz, Matthias Diebold, Alexander Kainz, Katharina A. Mayer, Konstantin Doberer, Nicolas Kozakowski, Philip F. Halloran, Georg A. Böhmig

**Affiliations:** ^1^ Division of Nephrology and Dialysis, Department of Medicine III, Medical University of Vienna, Vienna, Austria; ^2^ Clinic for Transplantation Immunology and Nephrology, University Hospital Basel, University of Basel, Basel, Switzerland; ^3^ Department of Pathology, Medical University of Vienna, Vienna, Austria; ^4^ Alberta Transplant Applied Genomics Centre, ATAGC, University of Alberta, Edmonton, AB, Canada

**Keywords:** antibody-mediated rejection, graft outcome, kidney transplantation, transcriptomics, transplant injury

## Abstract

Current knowledge about the factors correlating with functional decline and subsequent failure of kidney allografts in antibody-mediated rejection (ABMR) is limited. We conducted a cohort study involving 75 renal allograft recipients diagnosed with late ABMR occurring at least 6 months after transplantation. The study aimed to examine the correlation of molecular and histologic features with estimated glomerular filtration rate (eGFR) trajectories and death-censored graft survival. We focused on sum scores reflecting histologic ABMR activity versus chronicity and molecular scores of ABMR probability (ABMR_Prob_), injury-repair response (IRRAT) and fibrosis (ciprob). In multivariable Cox analysis, a Banff lesion-based chronicity index (ci+ct+cg[x2]; hazard ratio per interquartile range [IQR]: 1.97 [95% confidence interval: 0.97 to 3.99]) and IRRAT (1.93 [0.96 to 3.89]) showed the strongest associations with graft failure. Among biopsy variables, IRRAT exhibited the highest relative variable importance and emerged as the sole independent predictor of eGFR slope (change per IQR: −4.2 [−7.8 to −0.6] mL/min/1.73 m^2^/year). In contrast, morphologic chronicity associated with baseline eGFR only. We conclude that the extent of molecular injury is a robust predictor of renal function decline. Transcriptome analysis has the potential to improve outcome prediction and possibly identify modifiable injury, guiding targeted therapeutic interventions.

## Graphical Abstract

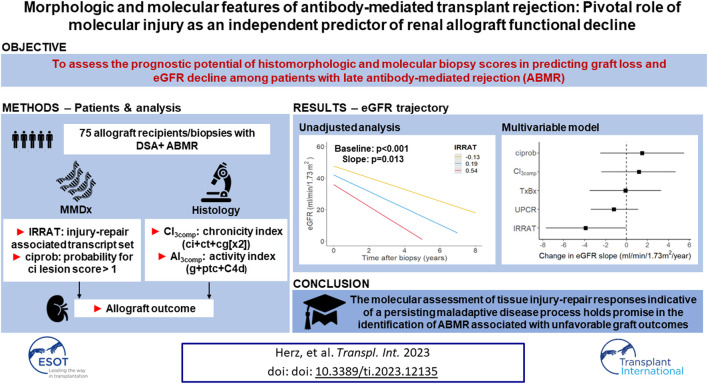

## Introduction

Antibody-mediated rejection (ABMR) is a cardinal cause of graft failure, characterized by a progressive decline in renal function and ultimately leading to accelerated graft loss^.^ [1–5]. Currently, there is only weak evidence supporting the effectiveness of any specific ABMR treatment [6]. According to the Banff scheme, the diagnosis of ABMR depends on certain combinations of distinct morphologic lesions, such as peritubular capillaritis (ptc), glomerulitis (g), glomerular double contours (cg), intimal arteritis (v) and/or vascular fibrous intimal thickening (cv), in conjunction with the detection of circulating donor-specific anti-HLA antibodies (DSA) and/or capillary C4d [7]. ABMR is, based on the presence or absence of features indicating rejection activity or chronic tissue injury, classified into different phenotypes, that are, active, chronic active, and chronic (inactive) ABMR [7].

For clinical practice, it would be highly beneficial to identify precise predictors of graft performance in the context of ABMR diagnosis, particularly features related to the dynamics of renal functional decline (rather than solely the estimated glomerular filtration rate [eGFR] at baseline), which could inform individualized clinical decisions and guide anti-rejection treatment. To date, however, only few studies have specifically addressed the predictive value of clinical and/or biopsy-based variables among ABMR patients. For instance, in a multicenter study involving 91 patients with chronic active ABMR, only a few factors such as study site, donor age, and HLA DSA class were found to be predictive of eGFR at the time of biopsy [4]. Surprisingly, none of the tested factors independently predicted eGFR slope, which itself emerged as a robust surrogate of graft survival [4]. Second, in a study of 70 ABMR patients conducted at our unit, graft survival was found to be associated with cg, while the only biopsy-based predictor of eGFR slope was the diagnosis of concurrent glomerulonephritis [8]. In a study of 278 patients with active ABMR, Viglietti et al. [9] established a dynamic composite prediction score integrating various factors, such as eGFR and interstitial fibrosis/tubular atrophy at the time of diagnosis, along with changes in eGFR, peritubular capillaritis, and DSA levels post-treatment. This score showed favorable calibration and discrimination, a finding that was validated in an independent cohort [9]. Finally, a recent study examining 147 ABMR cases, focusing on morphologic indices similar to those proposed for lupus nephritis [10], revealed that a chronicity index (CI) comprising cg, interstitial fibrosis (ci), tubular atrophy (ct), and cv was strongly predictive of graft survival, even independent of baseline eGFR [11]. However, a sum score incorporating a set of morphologic features reflecting ABMR activity (g, ptc, v, C4d) did not show the same impact [11]. Notably, analyses of eGFR trajectories were not included, which may be a crucial aspect to consider, as different (modifiable or non-modifiable) predictors of graft survival, may have differing impacts on eGFR intercept versus slope.

Incorporating gene expression analysis, e.g., using the Molecular Microscope® Diagnostic System (MMDx), alongside conventional histopathology, shows promising potential for enhancing outcome prediction [12]. In the INTERCOMEX multicenter study, a distinct pathogenesis-based transcript (PBT) set reflecting injury-repair response (IRRAT score) demonstrated the highest predictive value for graft survival [13]. Notably, its impact was even independent of morphologic features [13]. However, rates of eGFR decline, which reflect the actual dynamics of graft deterioration, were not analyzed.

This retrospective single-center study, conducted on late DSA-positive ABMR cases using the Vienna MMDx biopsy database, aimed to analyze the relative importance and independent predictive value of biopsy features. In addition to studying graft failure as an endpoint, a distinct aspect of our present study was the examination of dynamic changes in eGFR over time to gain a deeper understanding of how individual predictors impact the progression of rejection and graft dysfunction. Specifically, the study examined features reflecting the extent of rejection activity and acute versus chronic injury, by integrating gene expression analysis with morphologic results.

## Materials and Methods

### Study Design and Patients

**FIGURE 1:**
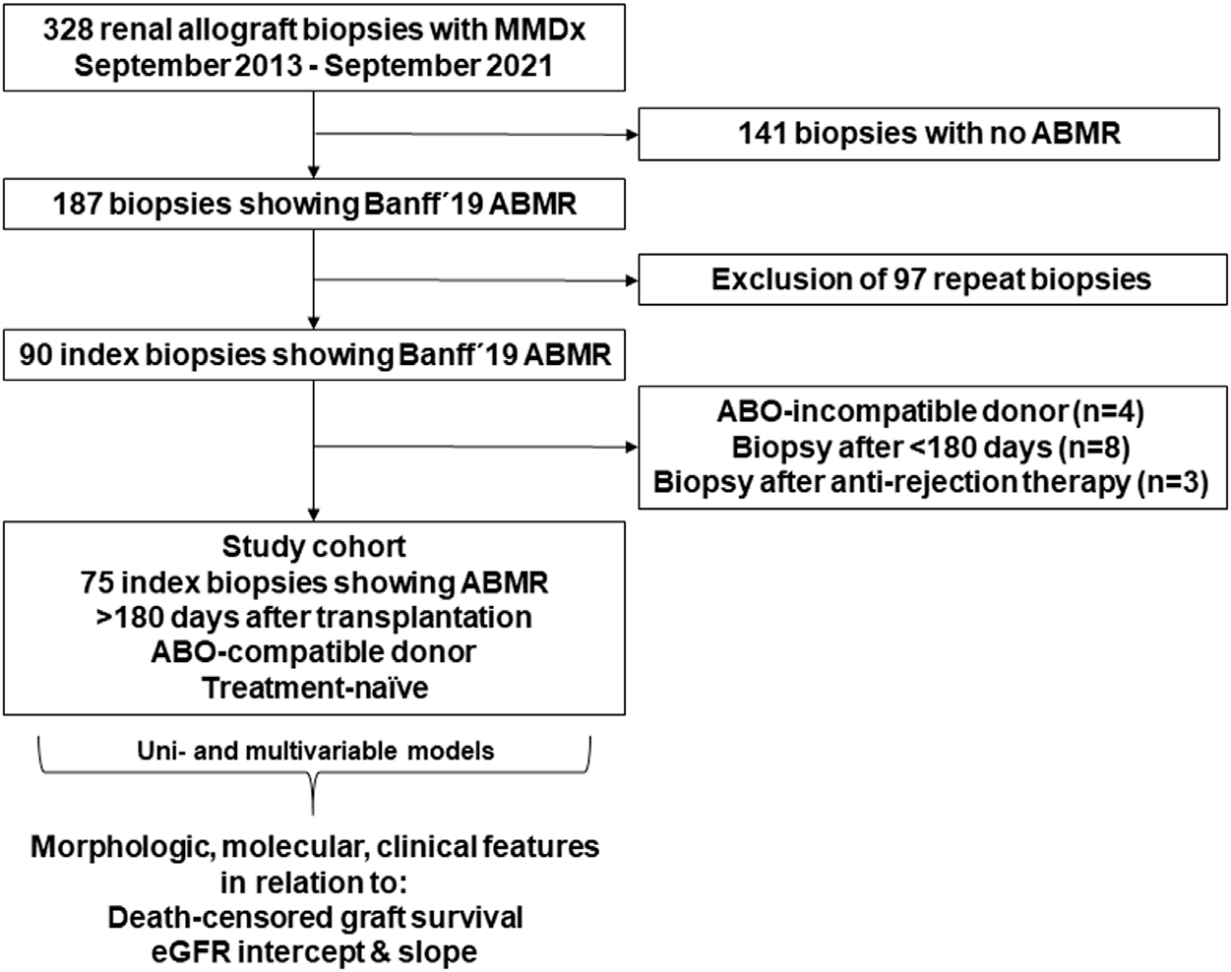
Flowchart illustrating the selection process of the study cohort. Key inclusion criteria were the availability of complete gene expression analysis via the Molecular Microscope® Diagnostic System (MMDx), diagnosis of antibody-mediated rejection (ABMR) according to the Banff 2019 scheme, and a treatment-naïve index biopsy after >180 days post-transplantation. From a total of 328 biopsies, 75 index biopsies performed in 75 recipients were selected for the study. Morphologic, molecular, and clinical variables were analyzed in relation to death-censored graft survival and estimated glomerular filtration rate (eGFR) intercept and slope.

This retrospective cohort study conducted at the transplant unit of the Medical University of Vienna included 75 recipients of an ABO-compatible renal allograft diagnosed with ABMR >180 days after transplantation. Study patients were selected from a cohort of 195 consecutive recipients who underwent at least one biopsy between September 2013 and September 2021, and for whom gene expression analysis via the MMDx platform was available (Figure 1). Baseline variables are provided in Tables 1, 2. For survival analysis, patient records were reviewed until March 2023. In addition, eGFR trajectories were determined by analyzing every creatinine measurement between 30 days before the biopsy and either death-censored graft failure (DCGF) or loss to follow-up. The study was approved by the institutional review board of the Medical University Vienna (approval number: 1451/2023).

**TABLE 1:** Baseline variables.

Variables	Study patients *n* = 75	Data available (n)
Variables recorded at the time of transplantation		
Recipient age, median (IQR)	45 (34.5–53.5)	75
Female sex, n (%)	37 (49.3)	75
Deceased donor, n (%)	63 (84.0)	75
Living donor, n (%)	12 (16.0)	75
Donor age (years), median (IQR)	46 (26–58)	73
Prior kidney transplant, n (%)	27 (37.0)	73
Current CDC panel reactivity ≥10%, n (%)	15 (21.4)	70
Preformed anti-HLA DSA, n (%)^a^	25 (62.5)	40
Cold ischemia time (hours), median (IQR)	12 (8.1–17.1)	71
HLA mismatch (A, B, DR), median (IQR)	3 (2–4)	73
HLA mismatch (A, B, C, DRB1, DQB1), median (IQR)	5 (4–6)	58
Initial immunosuppression		75
Induction with anti-thymocyte globulin, n (%)	31 (41.3)	75
Induction with IL-2 receptor antibody, n (%)	19 (25.3)	75
Tacrolimus-based immunosuppression, n (%)	44 (58.7)	75
Cyclosporine A-based immunosuppression, n (%)	29 (38.7)	75
mTOR inhibitor-based immunosuppression, n (%)	5 (6.7)	75
Peri-transplant immunoadsorption, n (%)	27 (36)	75
Variables recorded at the time of index biopsy		
Years after transplantation, median (IQR)	5.17 (2.41–13.21)	75
Renal parameters		
eGFR (mL/min/1.73 m^2^), median (IQR)	39.9 (26.7–59.6)	75
UPCR (mg/g), median (IQR)	373 (134–1252)	75
UPCR >1,000 mg/g, n (%)	23 (30.7)	75
Immunosuppression at the time of index biopsy		
Triple immunosuppression (%)	60 (80.0)	75
Tacrolimus, n (%)	51 (68.0)	75
Cyclosporine A, n (%)	20 (26.7)	75
mTOR inhibitor, n (%)	3 (4.0)	75
Belatacept, n (%)	1 (1.3)	75
Anti-rejection therapy triggered by ABMR diagnosis, n (%)	42 (56.0)	75
Bortezomib	20 (26.7)	75
Clazakizumab	15 (20.0)	75
BIVV009	9 (12.0)	75
Tocilizumab	2 (2.7)	75
Imlifidase/IVIG/rituximab	1 (1.3)	75
Immunoadsorption	1 (1.3)	75

DSA, donor-specific antibody; CDC, complement-dependent cytotoxicity; eGFR, estimated glomerular filtration rate; IQR, interquartile range; mTOR, mammalian target of rapamycin; UPCR, urinary protein/creatinine ratio.

^a^ For recipients transplanted before 2009, solid-phase HLA antibody screening on the transplant wait list was not available.

**TABLE 2:** Serologic data and biopsy results.

Variables	Cohort	Data (n)
DSA characteristics		
DSA-positive, n (%)	75 (100)	75
HLA class I DSA	45 (63.4)	71
HLA class II DSA	60 (84.5)	71
HLA class I plus II DSA	34 (47.9)	71
DSA-MFI^a^ >10,000	31 (42.5)	73
Morphologic biopsy results		
ABMR phenotypes, n (%)		
Active ABMR	15 (20)	75
Chronic active ABMR	47 (62.7)	75
Chronic (inactive) ABMR	13 (17.3)	75
Peritubular capillary C4d deposition	30 (40.0)	75
Single lesion scores, median (IQR)		
Capillary C4d (c4d)	0 (0 to 2)	75
Glomerulitis (g)	2 (1 to 2)	72
Peritubular capillaritis	1 (0 to 2)	75
Intimal arteritis (v)	0 (0 to 0)	62
Glomerular double contours (cg)	1 (0 to 2)	72
Interstitial fibrosis (ci)	2 (1 to 3)	75
Tubular atrophy (ct)	1 (1 to 2)	75
Vascular fibrous intimal thickening (cv)	1 (1 to 2)	62
Sum scores, median (IQR)		
AI (g+ptc+v+C4d)	4 (2 to 5)	61
AI_3comp_ (g+ptc+C4d)	4 (3 to 5)	72
CI (ci+ct+cv+[cgx2])	7 (4 to 10)	61
CI_3comp_ (ci+ct+[cgx2])	6 (3 to 8)	72
Banff borderline lesion, n (%)	4 (5.3)	75
Mixed rejection, n (%)	2 (2.7)	75
BK virus nephropathy, n (%)	1 (1.3)	75
Glomerulonephritis, n (%)^b^	3 (4.0)	75
Molecular biopsy results (MMDx)		
Rejection-associated scores, median (IQR)		
ABMR_Prob_ ^c^	0.54 (0.32 to 0.73)	75
TCMR	0.03 (0.02 to 0.05)	75
“all Rejection” score	0.67 (0.44 to 0.82)	75
Injury-associated scores, median (IQR)		
IRRAT	0.19 (−0.13 to 0.54)	75
ciprob	0.58 (0.30 to 0.75)	75
Most probable archetype, n (%)		
No rejection	15 (20)	75
TCMR	1 (1.3)	75
Early-stage ABMR	20 (26.7)	75
Fully-developed ABMR	31 (41.3)	75
Late-stage ABMR	8 (10.7)	75

ABMR, antibody-mediated rejection; AI, activity index; CI, chronicity index; ciprob, molecular classifier reflecting the probability of histologic ci lesion score >1; DSA, donor-specific antibody; IQR, interquartile range; IRRAT, transcript set associated with injury-repair response; MFI, mean fluorescence intensity; TCMR, T cell-mediated rejection.

^a^ MFI of the immunodominant DSA.

^b^ Cases of glomerulonephritis included two cases of IgA nephropathy and one case of unspecified immune complex-mediated glomerulonephritis.

^c^ Sixty-three recipients (84%) had an ABMR_Prob_ score >0.2.

### Biopsies

A total of 75 allograft biopsies were included in the study, performed for graft dysfunction, proteinuria and/or a positive DSA result. Morphologic analysis was performed on formalin-fixed paraffin-embedded sections. Single lesions and rejection phenotypes were scored and classified according to the Banff 2019 scheme [7]. We used published algorithms to calculate morphologic sum scores reflecting ABMR activity (activity index [AI]: g+ptc+v+C4d) and chronic injury (chronicity index [CI]: ci+ct+cv+[cgx2]) [11]. In 12 biopsies, the absence of arteries made it impossible to score vascular lesions. To ensure a larger sample size for statistical analysis, we simplified AI and CI (AI_3comp_; CI_3comp_) by excluding v and cv scores, respectively. However, for three biopsies, these indices could not be calculated due to an insufficient number of glomeruli for valid g and cg scoring. Notably, none of the biopsies showed significant v lesions, so v was not included individually in statistical models.

All index biopsies underwent gene expression analysis via the MMDx platform (ATAGC, University of Alberta, Edmonton, AB, Canada) [12]. Approximately 3 mm portions of one core from each biopsy underwent microarrays. Molecular scores were generated based on lesion-based transcript sets associated with rejection types [ABMR, T cell-mediated rejection (TCMR), “all Rejection”] and transcript sets related to injury-repair response (IRRAT score) or the probability of a histologic ci-lesion score >1 (ciprob score). Rejection archetypes were generated as described previously. The algorithms utilized a reference set of 1529 biopsies [14].

### HLA Antibody Detection

For HLA antibody detection, we utilized LABscreen Single Antigen assays (One Lambda, a brand of Thermo Fisher Scientific, Canoga Park, CA, USA). Serum samples were treated with ethylenediaminetetraacetic acid or subjected to heat inactivation to prevent complement interference [15]. The presence of DSA (mean fluorescence intensity [MFI] threshold >1,000) was determined according to serological and/or low- or high-resolution donor/recipient HLA typing (HLA-A, -B, -Cw, -DR, -DQ and/or DP).

### Immunosuppression

Out of the 75 biopsies, 9 were performed during routine clinical assessments, while 66 were conducted as part of screening for interventional trials. These trials evaluated different treatments, including bortezomib vs. placebo (ClinicalTrials.gov: NCT01873157; *n* = 50) [16], anti-interleukin-6 antibody clazakizumab (NCT03444103; *n* = 12) [17], imlifidase together with intravenous immunoglobulin/rituximab (NCT03897205; *n* = 1) or anti-C1s antibody BIVV009 (NCT02502903; *n* = 3) [18]. Details regarding baseline immunosuppression and treatment administered after the diagnosis of ABMR are provided in Table 1. Following index biopsies, 42 (56%) received antirejection therapy, which included investigational drugs or center-specific standard-of-care treatment, such as immunoadsorption (Table 1).

### Statistical Analysis

For descriptive analysis, continuous variables were reported as median (interquartile range [IQR]) and categorical variables as absolute counts and relative frequencies. For Kaplan-Meier survival analysis, variables were dichotomized based on their respective medians. Differences between groups were assessed using the log-rank test. Cox regression was used for univariable and multivariable survival analysis. Hazard ratios (HR) were reported per IQR increases of the tested variables. The proportional hazards assumption was assessed visually by plotting Schoenfeld residuals against time. To evaluate the functional form of the independent variables, they were fitted with restricted cubic splines with three knots. Then log hazards were plotted against the respective independent variables and deviations from linearity were visually assessed. Urinary protein/creatinine ratio (UPCR) was subsequently log-transformed. For eGFR slope analysis, we retrieved every serum creatinine measurement from 30 days before index biopsies until December 2022 from our database. Estimated GFR was calculated using the Chronic Kidney Disease Epidemiology Collaboration (CKD-EPI) equation [19]. Overall, 3885 measurements (in median 49 per patient (IQR: 38–64) were recorded. To examine associations between predictor variables and eGFR trajectories, we employed linear mixed models with eGFR as outcome variable and random slopes as well as random intercepts for the association between time and eGFR for each patient (random effect) using an unstructured variance-covariance matrix. Each predictor variable was included as main effect and in an interaction term with time. We used random forest analysis to calculate the relative importance of variables in relation to eGFR slope and graft loss, employing the permutation method. Statistical differences were tested at a two-sided significance level of 5%. All analyses were performed using R version 4.2.3 (R Foundation for Statistical Computing, Vienna, Austria). Utilized packages are provided as Supplemental Material.

## Results

### Patient and Biopsy Cohort

The study included 75 renal allograft recipients diagnosed with ABMR >180 days after transplantation. As detailed in Table 1, the cohort included 37 (49%) female patients, 27 (37%) recipients of a re-transplant, and 12 (16%) recipients of a living donor allograft. At the time of transplantation, the median recipient age was 45 years. Fifteen subjects (21.4%) had a cytotoxic panel reactivity ≥10% and 63% of the patients had preformed DSA. Twenty-seven (36%) subjects had been subjected to immunoadsorption-based desensitization [20]. Index biopsies were performed after a median of 5.17 years post-transplantation. Most patients (80%) were on triple immunosuppression, primarily tacrolimus-based therapy (68%). The median eGFR was 39.9 mL/min/1.73 m^2^, and median UPCR levels were 373 mg/g. After ABMR diagnosis, 56% of the patients received anti-rejection treatment, mostly in the context of interventional trials (Table 1). As shown in Table 2, all patients were DSA-positive at biopsy, with 60 patients (85%) having HLA class II DSA. The MFI of the immunodominant DSA was >10,000 in 43% of the patients. Biopsy results are provided in Table 2. Morphologic ABMR phenotypes included active ABMR (20%), chronic active ABMR (63%), and chronic (inactive) ABMR (17%), respectively. Thirty index biopsies (40%) were positive for C4d. In MMDx analysis, 63 (84%) specimens were classified as ABMR with an ABMR_Prob_ score ≥0.2, and 59 (78.7%) biopsies were grouped into one of three distinct morphological ABMR archetypes (Table 2). Among the 13 patients with morphologic chronic (inactive) ABMR, 8 recipients displayed an ABMR score equal to or above a threshold of 0.2. The most probable corresponding molecular archetypes for these cases were no rejection (*n* = 8), early stage ABMR (*n* = 1), fully-developed ABMR (*n* = 3) and late-stage ABMR (*n* = 1), respectively. Differences between morphologic ABMR phenotypes regarding morphologic and molecular indices/scores are detailed in Supplementary Table S1.

### Biopsy Results and Graft Survival

**FIGURE 2:**
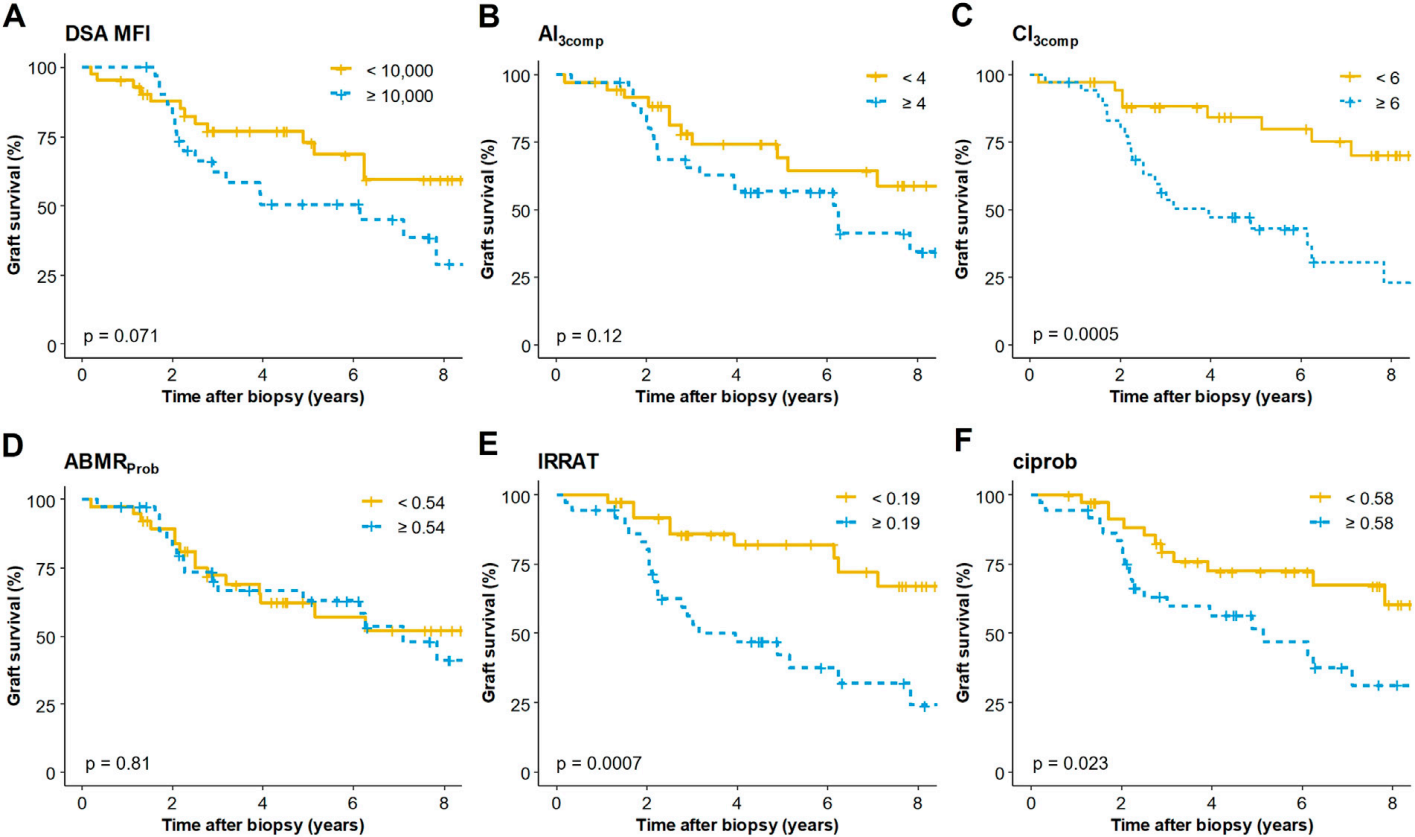
Kaplan Meier death-censored graft survival in relation to **(A)** the mean fluorescence intensity (MFI) of the immunodominant donor-specific antibody (DSA), **(B)** a simplified activity index (AI_3comp_), **(C)** a simplified chronicity index (CI_3comp_), **(D)** a classifier reflecting the probability of ABMR diagnosis (ABMR_Prob_), **(E)** an injury-repair response–associated transcript set (IRRAT) and **(F)** a classifier reflecting fibrosis (ciprob). Variables were dichotomized by their medians. The Mantel Cox log-rank test was used to compare survival rates between groups.

During follow-up, 32 episodes of DCGF were recorded, resulting in a median graft survival of 7.1 years. In a first step, we evaluated associations of biopsy features and clinical variables with DCGF applying unadjusted Cox proportional hazards analysis (Table 3). Among Banff single lesion scores, only cg and ct turned out to be associated with survival. When assessing morphologic indices that reflect either ABMR activity or chronic tissue injury, we observed strong associations for CI and CI_3comp_, but not for AI and AI_3comp_, respectively. Regarding molecular scores, we found IRRAT and ciprob scores, but not ABMR_Prob_, to be significant (Table 3). Among clinical variables, eGFR and UPCR recorded at the time of biopsy, showed a strong association with survival in the unadjusted analysis. Moreover, we found a trend for DSA-MFI >10,000, but no significant effects were observed for time to biopsy, HLA mismatch and donor age. Finally, the use of anti-rejection treatment did not show a significant association with improved survival (Table 3). Figure 2 depicts Kaplan-Meier graft survival curves for DSA-MFI and selected biopsy scores (AI_3comp_, CI_3comp_, ABMR_Prob_, IRRAT, ciprob) dichotomized by their median.

**TABLE 3:** Biopsy results and DCGF - Unadjusted Cox proportional hazards analysis.

Variables^a^	Hazard ratio (95% confidence interval)	*p*-value	Data (n)
Biopsy variables			
Morphologic single lesion scores			
C4d (c4d)	1.58 (0.91–2.74)	0.102	75
Glomerulitis (g)	0.88 (0.62–1.24)	0.46	72
Peritubular capillaritis (ptc)	1.17 (0.57–2.41)	0.67	75
Glomerular double contours (cg)	2.72 (1.39–5.33)	0.004	73
Interstitial fibrosis (ci)	1.75 (0.88–3.50)	0.11	75
Tubular atrophy (ct)	1.82 (1.19–2.78)	0.006	75
Vascular fibrous intimal thickening (cv)	1.00 (0.67–1.49)	>0.99	63
Morphologic indices			
AI (g+ptc+v+C4d)	1.39 (0.67–2.87)	0.38	61
AI_3comp_ (g+ptc+C4d)	1.20 (0.76–1.89)	0.43	73
CI (ci+ct+cv+[cgx2])	2.83 (1.24–6.43)	0.013	61
CI_3comp_ (ci+ct+[cgx2])	2.90 (1.51–5.57)	0.001	73
Molecular scores			
ABMR_Prob_	0.94 (0.52–1.68)	0.83	75
IRRAT	2.66 (1.56–4.55)	<0.001	75
ciprob	2.71 (1.32–5.54)	0.006	75
Clinical/immunological variables			
Variables recorded at transplantation			
Recipient age (years)	0.50 (0.31–0.81)	0.005	75
Male sex	0.87 (0.43–1.77)	0.71	75
Deceased donor	0.55 (0.24–1.23)	0.14	75
Donor age (years)	0.98 (0.50–1.95)	0.96	75
Prior kidney transplant	1.15 (0.54–2.45)	0.72	73
HLA mismatch (A, B, DR)	0.79 (0.42–1.49)	0.47	73
HLA mismatch (A, B, C, DRB1, DQB1)	0.73 (0.44–1.21)	0.22	58
Variables recorded at index biopsy			
Time to biopsy (years)	1.54 (0.93–2.55)	0.095	75
eGFR (ml/min/1.73m^2^)	0.23 (0.11–0.48)	<0.001	75
UPCR at biopsy (mg/g)	2.47 (1.33–4.60)	0.004	75
DSA MFI ≥10000	1.93 (0.93–3.98)	0.076	73
Tacrolimus-based immunosuppression	1.21 (0.56–2.64)	0.63	75
Anti-rejection treatment	0.83 (0.41–1.67)	0.60	75

ABMR, antibody-mediated rejection; AI, activity index; cg, glomerular double contours; CI, chronicity index; ci, interstitial fibrosis; ciprob, molecular classifier reflecting the probability of histologic ci lesion score >1; ct, tubular atrophy; cv, intimal fibrous thickening; DCGF, death-censored graft survival; DSA, donor-specific antibody; eGFR, estimated glomerular filtration rate; g, glomerulitis; IRRAT, transcript set associated with injury-repair response; MFI, mean fluorescence intensity; ptc, peritubular capillaritis; UPCR, urinary protein/creatinine ratio; v, intimal arteritis.

^a^ For continuous and ordinal categorical variables, hazard ratios were calculated per increase from the first to the third quartile.

In adjusted Cox proportional hazards analysis that included biopsy variables showing associations (*p* < 0.05) in univariable analysis, IRRAT score (HR per IQR: 1.93 [95% CI: 0.96 to 3.89], *p* = 0.067) and CI_3comp_ (1.97 [0.97 to 3.99], *p* = 0.059) exhibited the strongest associations with DCGF (Table 4). In a second model that also included clinical variables, the associations of biopsy variables with DCGF were no longer significant. Only eGFR at biopsy, and to a lesser extent recipient age, remained as the only variables associated with DCGF (Table 4). As shown in Supplementary Table S2 and Supplementary Figure S1, similar results were obtained in models including CI instead of CI_3comp_ (61 instead of 72 included cases), even though, a multivariable model including biopsy variables only revealed a significant impact of the IRRAT score.

**TABLE 4:** Adjusted Cox proportional hazards analysis for the prediction of DCGF^a^.

Variables^b^	Hazard ratio (95% confidence interval)	*p*-value	Data (n)
Model 1 (biopsy variables)			72
IRRAT	1.93 (0.96–3.89)	0.067	
CI_3comp_ (ci+ct+[cgx2])	1.97 (0.97–3.99)	0.059	
ciprob	1.24 (0.54–2.83)	0.61	
Model 2 (biopsy and clinical variables)			72
IRRAT	1.44 (0.66–3.14)	0.36	
CI_3comp_ (ci+ct+[cgx2])	1.36 (0.64–2.86)	0.42	
ciprob	0.96 (0.42–2.19)	0.92	
Recipient age (years)	0.54 (0.31–0.95)	0.033	
eGFR (mL/min/1.73 m^2^)	0.32 (0.15–0.70)	0.005	
UPCR at Bx (mg/g)	1.87 (0.87–4.03)	0.11	

cg, glomerular double contours; ci, interstitial fibrosis; ciprob, molecular classifier reflecting the probability of histologic ci lesion score >1; ct, tubular atrophy; cv, intimal fibrous thickening; DCGF, death-censored graft survival; eGFR, estimated glomerular filtration rate; IRRAT, transcript set associated with injury-repair response; UPCR, urinary protein/creatinine ratio.

^a^ Adjusted models (model 1: biopsy variables; model 2: biopsy plus clinical variables) included variables (morphologic indices, molecular scores and/or clinical parameters) associated with DCGF, in univariable analysis (see Table 3).

^b^ For continuous variables and ordinal categorical variables, hazard ratios were calculated per increase from the first to the third quartile.

**FIGURE 3:**
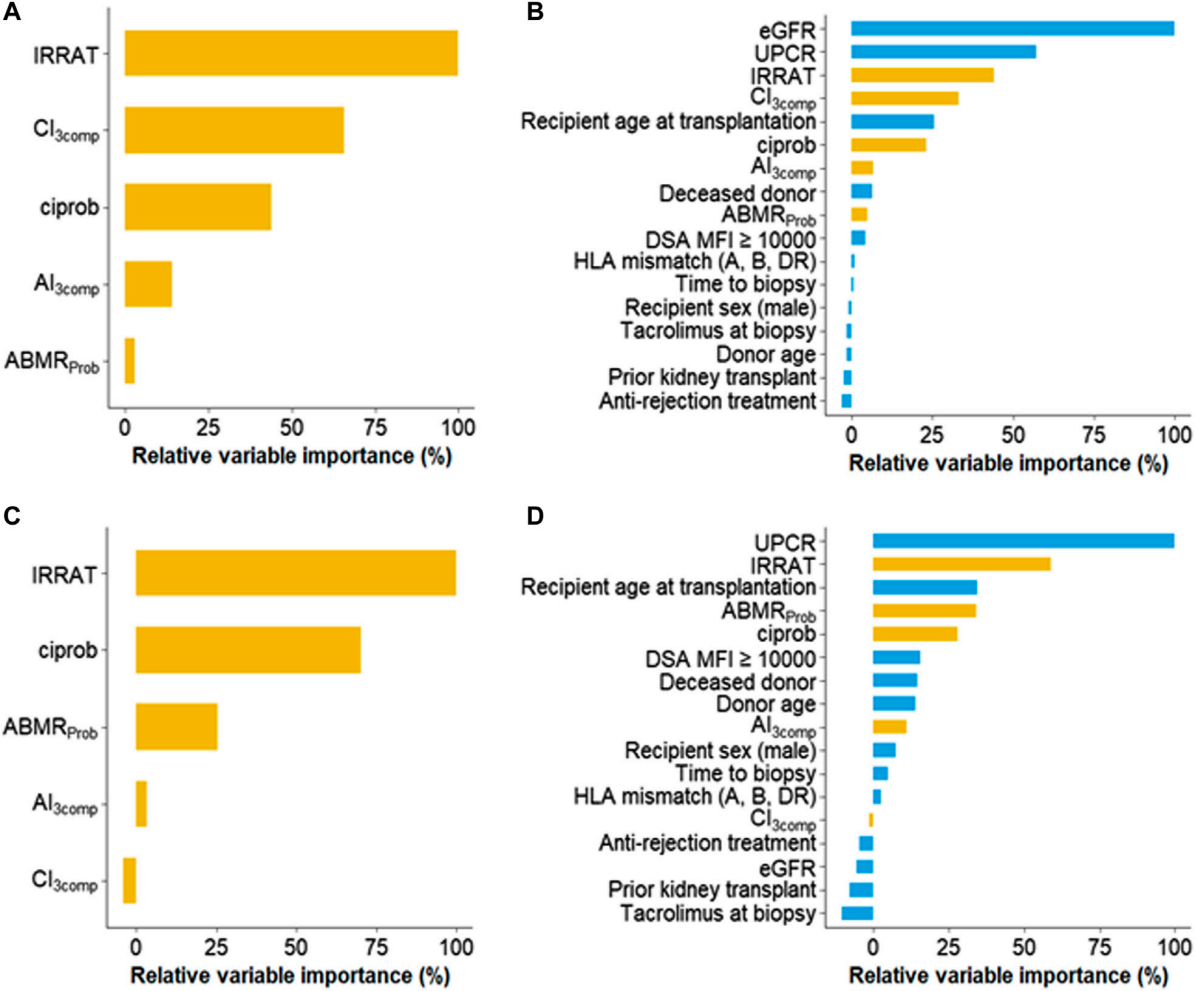
Random forest models to examine the impact of clinical, histologic, and molecular features on death censored graft loss **(A,B)** and estimated glomerular filtration rate (eGFR) slope **(C,D).** The prediction models comprised either biopsy-related features only **(A, C)** or a combination of both clinical and biopsy-related features **(B, D)**. Within each set, individual variables were sorted based on their importance. Abbreviations: ABMR_Prob_, molecular classifier reflecting the probability of histologic diagnosis of antibody-mediated rejection; AI_3comp_, simplified activity index (g+ptc+C4d); CI_3comp_, simplified chronicity index (ci+ct+cg[x2]); ciprob, molecular classifier reflecting the probability of histologic ci-lesion score >1; DSA, donor-specific antibody; eGFR, estimated glomerular filtration rate; IRRAT, injury-repair response-associated transcript; MFI, mean fluorescence intensity; UPCR, urinary protein/creatinine ratio.

In another approach, we applied random forest analysis to determine the relative importance of biopsy-based and/or clinical variables in predicting death-censored graft loss (Figure 3). In a first model evaluating biopsy parameters alone, IRRAT emerged as the most important variable, followed by features of chronic injury (CI_3comp_, ciprob). Morphologic or histologic ABMR activity had the least importance. In a second model including clinical variables, eGFR emerged as the most important variable, followed by UPCR, IRRAT, CI_3comp_ and recipient age (Figure 3). Similar results were obtained for unmodified CI and AI (Supplementary Figure S2).

### Biopsy Results in Relation to eGFR Slope

**FIGURE 4:**
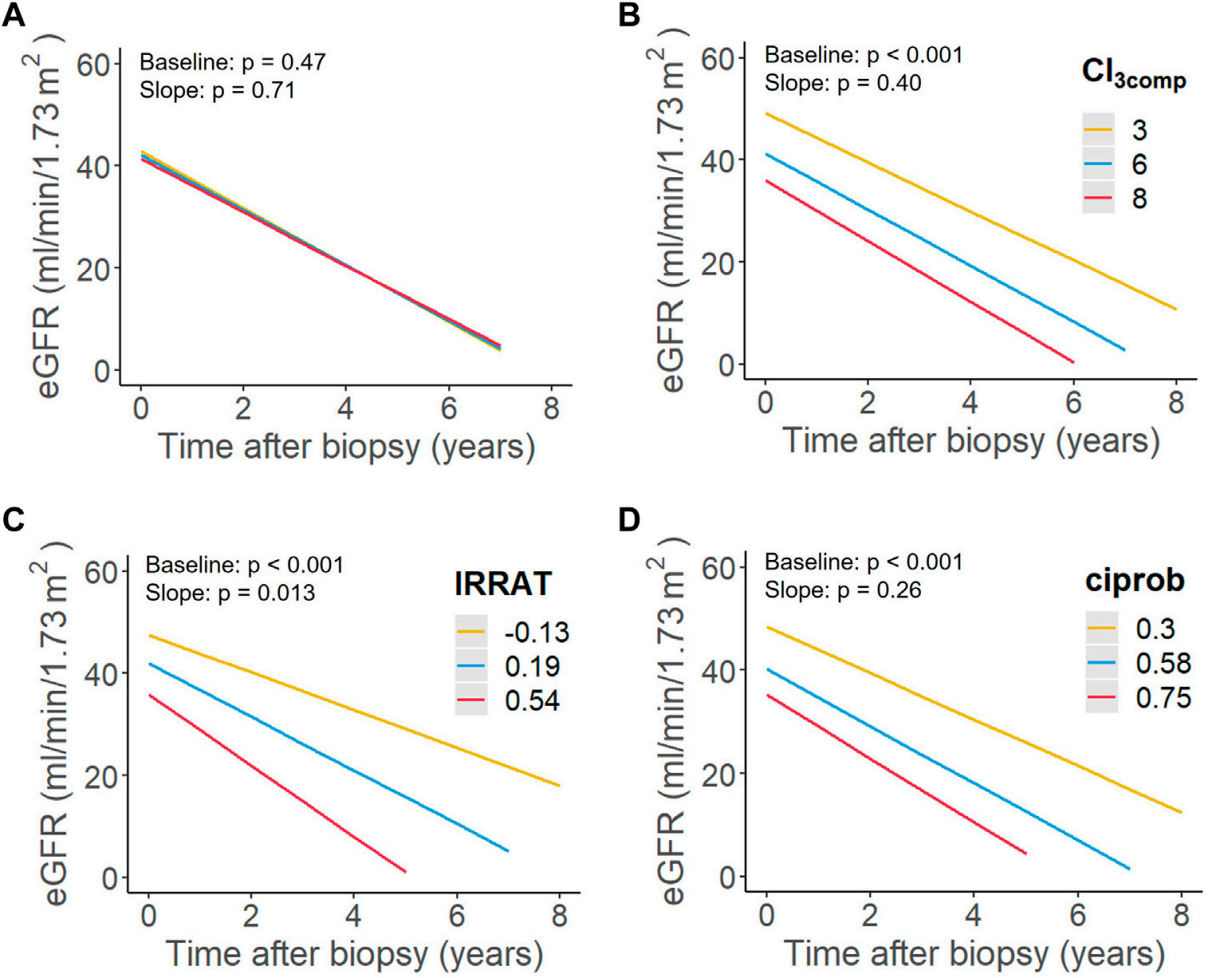
Predictive performance of **(A)** a simplified activity index (AI_3comp_), **(B)** a simplified chronicity index (CI_3comp_), **(C)** an injury-repair response–associated transcript set (IRRAT) as well as **(D)** a classifier reflecting fibrosis (ciprob) in relation to estimated glomerular filtration rate (eGFR) trajectory. Shown are the estimated association between each variable and eGFR slope in unadjusted linear mixed models. Lines were drawn for the first, second, and third quartile of each predictor.

In a linear mixed model, which included in median 49 eGFR values per subject (from 30 days before biopsy to DCGF or loss of follow-up), mean eGFR at baseline, the intercept, was 41.4 (95% confidence interval: 37.6–45.2) mL/min/1.73 m [2] and the mean slope was −5.4 (−7.0 to 3.7) mL/min/1.73 m [2] per year (data not shown). In unadjusted models, the IRRAT score was associated with lower eGFR at baseline and, as the only variable, with a steeper eGFR slope, while chronicity indices (CI, CI_3comp_) and ciprob were only associated with lower baseline eGFR values. Among clinical variables, time to biopsy and UPCR were associated with eGFR at baseline, the latter with a trend towards an association with eGFR slope (Table 5; Figure 4). In a multivariable model including biopsy variables showing associations (*p* < 0.05) in univariable analysis, IRRAT remained associated with eGFR slope. Conversely, CI_3comp_ remained associated with baseline eGFR (Table 5). Similar results were observed in a second model that adjusted also for clinical variables, even though the effects of IRRAT on eGFR slope were no longer significant (*p* = 0.066). Among clinical variables only time to biopsy exhibited a significant association with eGFR at baseline (Table 5). As shown in Supplementary Table S3 and Supplementary Figure S1, multivariable models including CI instead of CI_3comp_ revealed comparable results (61 instead of 72 included cases).

**TABLE 5:** Linear mixed models for the prediction of eGFR trajectories after index biopsy.

	Variables^a^ ^,^ ^b^		Baseline association (time = 0)	*p*-value	Change in slope (interaction term)	*p*-value	n
Unadjusted analysis	**Biopsy variables**						
	AI (g+ptc+v+C4d)		−1.8 (−8.1–4.6)	0.59	−0.2 (−2.9–2.5)	0.88	61
	AI_3comp_ (g+ptc+C4d)		−1.9 (−7.1–3.3)	0.47	0.4 (−1.9–2.8)	0.71	72
	CI (ci+ct+cv+[cgx2])		−14.6 (−20.6–−8.5)	<0.001	−0.5 (−3.4–2.3)	0.72	61
	CI_3comp_ (ci+ct+[cgx2])		−15.9 (−22.1–−9.6)	<0.001	−1.4 (−4.5–1.8)	0.40	72
	ABMR_Prob_		1.9 (−4.6–8.3)	0.58	1.5 (−1.3–4.3)	0.29	75
	IRRAT		−13 (−19–−7.1)	<0.001	−3.6 (−6.4–−0.9)	0.013	75
	ciprob		−14.1 (−20.4–−7.9)	<0.001	−1.8 (−4.9–1.3)	0.26	75
	**Clinical/immunological variables at transplantation**						
	Recipient age		2.5 (−2.6–7.6)	0.34	2.6 (0.5–4.7)	0.020	75
	Male sex		2.9 (−4.7–10.5)	0.46	−1.9 (−1.3–5.2)	0.25	75
	Deceased donor		0.4 (−10–−3.8)	0.95	2.9 (−1.5–7.4)	0.20	75
	Donor age		0.7 (−5.9–7.3)	0.83	1.3 (−1.5–4.2)	0.38	73
	Prior kidney transplant		−0.7 (−8.8–7.3)	0.86	−0.4 (−3.9–3)	0.81	73
	HLA mismatch (A, B, DR)		2.8 (−4.5–10.1)	0.45	0.7 (−2.4–3.8)	0.67	73
	HLA mismatch (A, B, C, DRB1, DQB1)		4.58 (−0.8–10)	0.10	0.8 (−1.8–3.4)	0.55	58
	**at index biopsy**						
	Time to biopsy (years)		−9.3 (−15.1–−3.6)	0.002	−1.1 (−3.7–1.5)	0.40	75
	UPCR at biopsy (mg/g)		−7.2 (−11.5–-3.0)	0.001	−1.8 (−3.8–0.1)	0.065	75
	DSA MFI ≥10000		1.9 (−6–9.8)	0.63	−2.7 (−6–0.6)	0.11	73
	Tacrolimus-based immunosuppression		5.6 (−2.5–13.6)	0.18	−1.3 (−4.8–2.2)	0.46	75
	Anti-rejection treatment		3 (−4.7–10.7)	0.44	−0.2 (−3.5–3.2)	0.93	75
Model 1 (biopsy variables)							72
	CI_3comp_ (ci+ct+[cgx2])		−11.2 (−17.5–−4.9)	<0.001	−0.1 (−3.4–3.2)	0.96	
	IRRAT		−5.8 (−12.7–1)	0.10	−4.2 (−7.8–−0.6)	0.029	
	ciprob		−7 (−14.1–0.2)	0.066	1.2 (−2.6–5)	0.55	
Model 2 (biopsy and clinical variables)							72
	CI_3comp_ (ci+ct+[cgx2])		−8.8 (−15.6–−2.1)	0.016	1.2 (−2.4–4.7)	0.54	
	IRRAT		−8.4 (−15.7–−1.1)	0.034	−3.9 (−7.7–0)	0.066	
	ciprob		−4.2 (−11.8–3.4)	0.30	1.5 (−2.5–5.5)	0.49	
	Recipient age		−2.5 (−7.1–2.2)	0.31	2 (−0.5–4.5)	0.14	
	Time to biopsy		−6.9 (−13.1–−0.6)	0.043	−0.1 (−3.5–3.3)	0.95	
	UPCR at biopsy		−1.1 (−5.3–3.1)	0.63	−1.2 (−3.4–1.1)	0.32	

AI, activity index; cg, glomerular double contours; CI, chronicity index; ci, interstitial fibrosis; ciprob, molecular classifier reflecting the probability of histologic ci lesion score >1; ct, tubular atrophy; cv, intimal fibrous thickening; DSA, donor-specific antibody; eGFR, estimated glomerular filtration rate; g, glomerulitis; IRRAT, transcript set associated with injury-repair response; MFI, mean fluorescence intensity; ptc, peritubular capillaritis; UPCR, urinary protein/creatinine ratio; v, intimal arteritis.

^a^ Each predictor is included as main effect and in an interaction term with time.

^b^ For continuous and ordinal categorical independent variables, the estimates are shown for an increase by one interquartile range of the respective variable.

In random forest models, irrespective whether clinical variables were included or not, the IRRAT score turned out to be the most important biopsy variable in predicting eGFR slope. Other biopsy features demonstrated lesser importance (ciprob) or showed negligible impact (features of ABMR activity). Among clinical variables, UPCR displayed the highest relative importance (Figure 3). IRRAT demonstrated a high level of variable importance also in models that incorporated CI and AI instead of CI_3comp_ and AI_3comp_ (Supplementary Figure S2).

## Discussion

A major finding of this study, which aimed to identify predictors of graft performance in late ABMR, was that among a selection of various morphologic and molecular biopsy features the MMDx-generated IRRAT score emerged as the sole independent predictor of dynamic eGFR decline. In a model adjusted for clinical variables this association lost statistical significance (*p* = 0.066), although the point estimate of the effect size remained consistent between the models, suggesting the absence of relevant effect modification by the included clinical variables. A considerable predictive power was supported by the fact that the IRRAT score exhibited the highest variable importance in random forest models. In contrast, Banff lesion-based or molecular scores reflecting chronic injury solely influenced baseline eGFR without affecting eGFR trajectories [11, 12]. Morphologic and molecular scores indicating ABMR activity or probability had no effect.

Our approach, which involved the use of linear mixed models incorporating a substantial number of creatinine measurements (a median of 49 measurements per patient), allowed us to examine associations for both baseline eGFR and eGFR slopes. In line with previous research [4, 8], we observed a significant decline in renal functional following ABMR diagnosis, with an average eGFR slope of −5.4 mL/min/1.73 m [2] per year. Through this detailed examination of the eGFR course, we were able to distinguish between processes contributing to the dynamic progression of graft dysfunction, which might be amenable to intervention, and processes related to the irreversible loss of nephrons. Both types of processes can associate with a shortened period of graft survival.

Among tested variables, we found that the IRRAT PBT set was the most powerful biopsy-derived predictor of eGFR decline. This finding remained significant even after adjusting for clinical variables such as recipient age, time to biopsy, and proteinuria, each of which individually showed significant associations. The changes in eGFR slope observed were substantial, with approximately −4 mL/min/1.73 m^2^/year decrease for each IQR increase in IRRAT. Our findings underscore the significance of integrating molecular gene expression analysis for predicting the risk of graft dysfunction and loss.

Injury-repair response–associated transcripts (IRRATs) were initially identified from early rejection-free post-transplant biopsies obtained within the first 6 weeks after transplantation by comparing biopsies with dysfunction to pristine protocol biopsies [21]. Unlike acute tubular injury based on morphological analysis, a pathogenesis-based transcript set generated from the 30 top IRRATs (IRRAT score) was found to correlate with eGFR at the time of biopsy and subsequent eGFR decline [21]. IRRATs comprise transcripts that are increased in acute kidney injury, such as kidney injury molecule 1 [22], and they were found to overlap substantially with injury and repair-induced transcripts triggered by the transplantation process in mouse kidney isografts [21]. In light of these results, our finding of IRRAT as a biopsy-based predictor of eGFR slope implies that repair responses, as evidenced by distinct transcriptional changes, may be maladaptive and insufficient to effectively counter ongoing parenchymal injury.

In a large multicenter trial (INTERCOMEX), the IRRAT score emerged as one of the strongest predictors of graft loss, in both patients with pure ABMR (*n* = 321) and those with any diagnostic category (*n* = 1,120), while rejection-related scores did not demonstrate relevant predictive value [13]. However, the impact of IRRAT on the course of eGFR during follow-up was not analyzed. Our present study aimed to address this gap and provide additional insights into the relationship between IRRAT score and both the baseline eGFR and its slope. Previous studies have demonstrated a close association between the eGFR slope in ABMR, serving as a potentially valuable surrogate endpoint, and long-term graft survival [4, 8]. As expected, our mixed model analysis revealed associations between IRRAT score and baseline eGFR, and univariable Cox regression demonstrated a strong association between IRRAT score and graft loss (2.7-fold risk; *p* < 0.001). However, in a multivariable Cox model that considered clinical variables such as eGFR, recipient age, and UPCR, the survival effect of the IRRAT score was no longer significant, with baseline eGFR emerging as the dominant predictor. These findings align with the major findings of INTERCOMEX, where random forest survival analysis identified baseline eGFR as one of the most important predictors of outcome [13]. Additionally, a recent multicenter study that focused on late DSA-positive ABMR found that eGFR at the time of biopsy was the sole predictor of graft survival [4].

Remarkably, established histomorphologic lesion scores reflecting ABMR activity, such as scores of single lesions reflecting inflammation in the microcirculation (g and ptc), did not exhibit predictive value for clinical outcomes in our cohort. Even when combining different single lesion scores (g, ptc, c4d, and/or v) to calculate activity indices, they still failed to demonstrate significant predictive capability. These findings align with a recent study by Haas et al. [11], further supporting the limited predictive value of these histomorphologic scores for clinical outcomes in the context of ABMR.

In the study by Haas et al. [11], however, a Banff-based histologic chronicity index incorporating ci, ct, cv, and cg, demonstrated predictive value for DCGF, even after adjusting for eGFR. In our cohort, the chronicity index (CI) or a simplified version excluding cv lesion scores, showed a significant effect in predicting DCGF in unadjusted analysis, but this association was no longer observed in multivariable analysis once clinical variables were considered. Several potential explanations could account for the differences between the two studies. One factor may be the smaller sample size in our cohort, which could have limited the statistical power for more complex analyses. Moreover, differences in selection criteria between the two studies could have contributed to the variations observed. Our cohort focused specifically on late ABMR cases, whereas the study by Haas et al. [11] included a significant number of early ABMR cases. Including only late ABMR cases, our study population exhibited significantly higher levels of chronic injury, as indicated by a median CI of 7 (IQR: 4–10). This contrasted with lower CI values observed in cases recruited from Cedars Sinai Medical Center, Los Angeles (3 [1–7]) and Necker Hospital, Paris (2 [0–4]) [23]. The timing of ABMR diagnosis may have major implications for outcome effects. In a recent analysis of the ANZDATA registry, which included 510 patients with early ABMR and 396 patients with late ABMR (defined as occurring >180 days after transplantation), late ABMR was associated with a twofold increased risk of graft loss, despite the utilization of various treatment approaches [24]. Underscoring treatment resistance of late ABMR, the use of different types of treatment in our cohort, both within and outside interventional trials, failed to improve eGFR slope or graft survival rates.

There are several inherent limitations of our study that should be acknowledged. Firstly, it is important to note that our study is a retrospective single-center evaluation with a partially confirmatory nature. While the multicenter INTERCOMEX trial has previously demonstrated a robust predictive value of IRRAT in relation to graft outcome, the strength of our present study, however, lies in its high granularity, encompassing detailed analyses of both biopsy-based and clinical endpoints, including comprehensive assessments of eGFR trajectories. Moreover, it is noteworthy that a significant proportion of our patients had preformed DSA and underwent desensitization, factors known to potentially influence outcome results. This could limit the generalizability of our findings to cohorts primarily consisting of patients with *de novo* DSA [25]. Another limitation to generalizability may arise from a heterogeneity in biopsy indications, including those performed in the context of interventional trials. In addition, our sample size was limited, resulting in insufficient statistical power to detect small effect sizes. Due to the risk of overfitting, we were unable to construct larger multivariable models. Another limitation was the lack of adequate arterial sections in 12 of the 75 index biopsies. This prevented us from calculating the original CI described in the study by Haas et al. [11] for all patients. To circumvent this caveat and thus to increase the sample size for statistical analysis, we decided to simplify the activity and chronicity indices to three variables each. This approach was supported by our observation that, unlike the findings in the study by Haas et al. [11], arterial intimal fibrous thickening indicated by the cv score was not associated with DCGF. Additionally, a recent study proposing an algorithm for clustering kidney biopsies based on their chronic Banff lesion scores found that ci, ct, and cg were the most informative lesions for outcome prediction, while the other including cv were less important [26]. Nonetheless, even when using the original indices and reducing the sample size to 61 subjects, the results remained largely unchanged. Our study highlights the common issue of sampling error in clinical practice and supports the use of molecular analysis, which may be less susceptible to sampling bias [27]. It is important to note that we specifically focused on a cohort selected for late ABMR. Hence, it remains unclear whether the IRRAT score is also useful for predicting eGFR slope in cases of early ABMR, where gene expression patterns related to injury could be confounded by transient perturbations such as ischemia reperfusion injury. Lastly, a potential limitation is the heterogeneity of therapeutic approaches in our cohort. However, the lack of any long-term treatment effect implies that this heterogeneity may not have had a significant impact on our outcome results, particularly regarding predictors that showed significance in univariable analysis. In this context, it is noteworthy that treatment in our patients was not guided by molecular features reflecting injury, such as IRRAT or ciprob.

In conclusion, our study provides evidence that a PBT set associated with injury-repair response (IRRAT) may have particular value in predicting eGFR decline in patients with late ABMR (diagnosed after >180 days after transplantation). Unlike morphologic and molecular features of chronic injury, which may indicate irreversible nephron loss and not necessarily correlate with accelerated functional decline after biopsy, injury-repair-associated transcripts reflect a potentially modifiable state of ongoing graft damage that is not visible with conventional morphology. Future trials, which may also include earlier types of ABMR, are needed to investigate whether changes in IRRAT score can be observed in response to effective ABMR therapy, potentially serving as a guide for targeted anti-rejection treatment. Additionally, it remains to be investigated whether patients with higher baseline IRRAT scores exhibit greater treatment responses compared to those with predominant chronic injury patterns.

## Data Availability

The data analyzed in this study is subject to the following licenses/restrictions: non-anonymized data. Requests to access these datasets should be directed to the corresponding author (GB).

## Abbreviations

ABMR, antibody-mediated rejection; ABMR_Prob_, molecular classifier reflecting the probability of histologic ABMR diagnosis; AI, activity index (g+ptc+v+C4d); AI_3comp_, simplified activity index (g+ptc+C4d); cg, glomerular double contours; CI, chronicity index (ci+ct+cv+cg[x2]); CI_3comp_, simplified chronicity index (ci+ct+cg[x2]); ci, interstitial fibrosis; ciprob, molecular classifier reflecting the probability of histologic ci-lesion score >1; CKD-EPI, Chronic Kidney Disease Epidemiology Collaboration; ct, tubular atrophy; cv, vascular fibrous intimal thickening; DCGF, death-censored graft failure; DSA, donor-specific antibody; eGFR, estimated glomerular filtration rate; g, glomerulitis; IQR, interquartile range; IRRAT, injury-repair response-associated transcript; MFI, mean fluorescence intensity; MMDx, Molecular Microscope® Diagnostic System; PBT, pathogenesis-based transcript set; ptc, peritubular capillaritis; TCMR, T cell-mediated rejection; UPCR, urinary protein/creatinine ratio; v, intimal arteritis.
