# Supplementary material for: Morphologic and Molecular Features of Antibody-Mediated Transplant Rejection: Pivotal Role of Molecular Injury as an Independent Predictor of Renal Allograft Functional Decline
Source: Transpl Int. 2023 Dec 19;36:12135. doi: 10.3389/ti.2023.12135 (PMC10758445; doi:10.3389/ti.2023.12135)
Supplement: Supplementary file 1 [file DataSheet1.pdf]

Supplemental Digital Content for:

Morphologic and molecular features of antibody-mediated transplant rejection: Pivotal role of molecular injury as an independent predictor of renal allograft functional decline

Carsten T. Herz[1], Matthias Diebold[1,2], Alexander Kainz[1], Katharina Mayer[1], Konstantin Doberer[1], Nicolas Kozakowski[3], Philip F Halloran[4], Georg A. Böhmig[1]

[1]*Division of Nephrology and Dialysis, Department of Medicine III, Medical University of Vienna, Vienna, Austria*
[2]*Clinic for Transplantation Immunology and Nephrology, University Hospital Basel, University of Basel, Basel, Switzerland*
[3]*Department of Pathology, Medical University of Vienna, Vienna, Austria*
[4]*Alberta Transplant Applied Genomics Centre, ATAGC, University of Alberta, Edmonton, AB Canada*

List of R Packages:

- **ggplot2:** H. Wickham. ggplot2: Elegant Graphics for Data Analysis. Springer-Verlag New York, 2016.
- **lme4:** Douglas Bates, Martin Maechler, Ben Bolker, Steve Walker (2015). Fitting Linear Mixed-Effects Models Using lme4. Journal of Statistical Software, 67(1), 1-48. doi:10.18637/jss.v067.i01.
- **lmerTest:** Kuznetsova A, Brockhoff PB, Christensen RHB (2017). “lmerTest Package: Tests in Linear Mixed Effects Models.” Journal of Statistical Software, *82*(13), 1-26. doi:10.18637/jss.v082.
- **randomForestSRC:** Ishwaran H. and Kogalur U.B. (2023). Fast Unified Random Forests for Survival, Regression, and Classification (RF-SRC), R package version 3.2.2.
- **rms:** Harrell Jr FE (2023). _rms: Regression Modeling Strategies_. R package version 6.7-0, <https://CRAN.R-project.org/package=rms>.
- **sjPlot:** Lüdecke D (2023). _sjPlot: Data Visualization for Statistics in Social Science_. R package version 2.8.14, <https://CRAN.R-project.org/package=sjPlot>.
- **survival:** Therneau T (2023)._A Package for Survival Analysis in R_. R package version 3.5-3, <https://CRAN.R-project.org/package=survival>.
- **survminer:** Kassambara A, Kosinski M, Biecek P (2021). _survminer: Drawing Survival Curves using 'ggplot2'_. R package version 0.4.9, <https://CRAN.R-project.org/package=survminer>.

Table S1. Comparison of morphologic and molecular scores/indices reflecting acute or chronic injury between ABMR phenotypes.

Variable[a]	Active ABMR (n=15)	Chronic active ABMR (n=47)	Chronic ABMR (n=13)	P value[b]
**AI_{3comp}**	4 (3.5 to 5)	4.5 (3.25 to 5)	1 (0.5 to 1)	<0.001
**CI_{3comp}**	2 (0.5 to 3.5)	8 (4.25 to 9)	4 (3 to 7)	<0.001
**IRRAT**	-0.02 (-0.11 to 0.2)	0.33 (0.02 to 0.58)	-0.14 (-0.39 to 0.17)	0.027
**ciprob**	0.4 (0.24 to 0.69)	0.63 (0.34 to 0.82)	0.53 (0.28 to 0.58)	0.081

ABMR, antibody-mediated rejection; AI_{3comp}, simplified activity index; CI_{3comp}, simplified chronicity index; ciprob, molecular classifier reflecting the probability of histologic ci lesion score >1; IRRAT, transcript set associated with injury-repair response.

[a]Data are presented as median and interquartile range.

[b]The Kruskal-Wallis test was employed for statistical inter-group comparisons.

Table S2. Adjusted Cox proportional hazards analysis for the prediction of DCGF using the original chronicity index[a]

Variables[b]	Hazard ratio (95% confidence interval)	*P* value	Data (n)
**Model 1 (biopsy variables)**			61
IRRAT	2.21 (1.04 to 4.70)	0.039	
CI (ci+ct+cv+[cgx2])	1.65 (0.68 to 4.02)	0.27	
ciprob	1.69 (0.63 to 4.50)	0.30	
**Model 2 (biopsy and clinical variables)**			61
IRRAT	1.71 (0.74 to 3.98)	0.21	
CI (ci+ct+cv+[cgx2])	1.06 (0.40 to 2.80)	0.91	
ciprob	1.28 (0.49 to 3.36)	0.62	
Recipient age (years)	0.50 (0.26 to 0.94)	0.033	
eGFR (ml/min/1.73m^2)	0.36 (0.15 to 0.89)	0.028	
UPCR at biopsy (mg/g)	1.87 (0.81 to 4.36)	0.15	

cg, glomerular double contours; CI, chronicity index; ci, interstitial fibrosis; ciprob, molecular classifier reflecting the probability of histologic ci lesion score >1; ct, tubular atrophy; cv, intimal fibrous thickening; DCGF, death-censored graft survival; eGFR, estimated glomerular filtration rate; IRRAT, transcript set associated with injury-repair response; UPCR, urinary protein/creatinine ratio.

[a]Adjusted models (model 1: biopsy variables; model 2: biopsy plus clinical variables) included variables (morphologic indices, molecular scores and/or clinical parameters) associated with DCGF in univariable analysis (see Table 3).

[b]For continuous variables and ordinal categorical variables, hazard ratios were calculated per increase from the first to the third quartile.

Table S3: Linear mixed models for the prediction of eGFR trajectories after index biopsy using the original chronicity index

	Variables[a,b]	Baseline association (time = 0)	*P* value	Change in slope (interaction term)	*P* value	N
**Model 1 (biopsy variables)**						
	CI (ci+ct+cv+[cgx2])	-10.8 (-16.8 to -4.8)	0.001	0.4 (-2.4 to 3.2)	0.79	61
	IRRAT	-5.3 (-12.1 to 1.4)	0.14	-5.1 (-8.3 to -1.8)	0.005	
	ciprob	-6.8 (-14.3 to 0.8)	0.088	2.0 (-1.6 to 5.6)	0.29	
**Model 2 (biopsy and clinical variables)**						61
	CI (ci+ct+cv+[cgx2])	-8.0 (-14.3 to -1.7)	0.021	1.4 (-1.7 to 4.4)	0.39	
	IRRAT	-8.1 (-15.2 to -1.1)	0.035	-5.1 (-8.4 to -1.6)	0.008	
	ciprob	-4.3 (-12.0 to 3.4)	0.30	2.8 (-0.9 to 6.5)	0.17	
	Recipient age	-4.8 (-9.8 to 0.2)	0.076	2.0 (-0.5 to 4.5)	0.14	
	Time to biopsy	-8.5 (-14.8 to -2.1)	0.016	-0.8 (-3.9 to 2.3)	0.63	
	UPCR at biopsy	-1.5 (-5.6 to 2.6)	0.50	-0.7 (-2.7 to 1.3)	0.50	

cg, glomerular double contours; CI, chronicity index; ci, interstitial fibrosis; ciprob, molecular classifier reflecting the probability of histologic ci lesion score >1; ct, tubular atrophy; cv, intimal fibrous thickening; eGFR, estimated glomerular filtration rate; IRRAT, transcript set associated with injury-repair response; UPCR, urinary protein/creatinine ratio.

[a]Each predictor is included as main effect and in an interaction term with time.

[b]For continuous and ordinal categorical independent variables, the estimates are shown for an increase by one interquartile range of the respective variable.

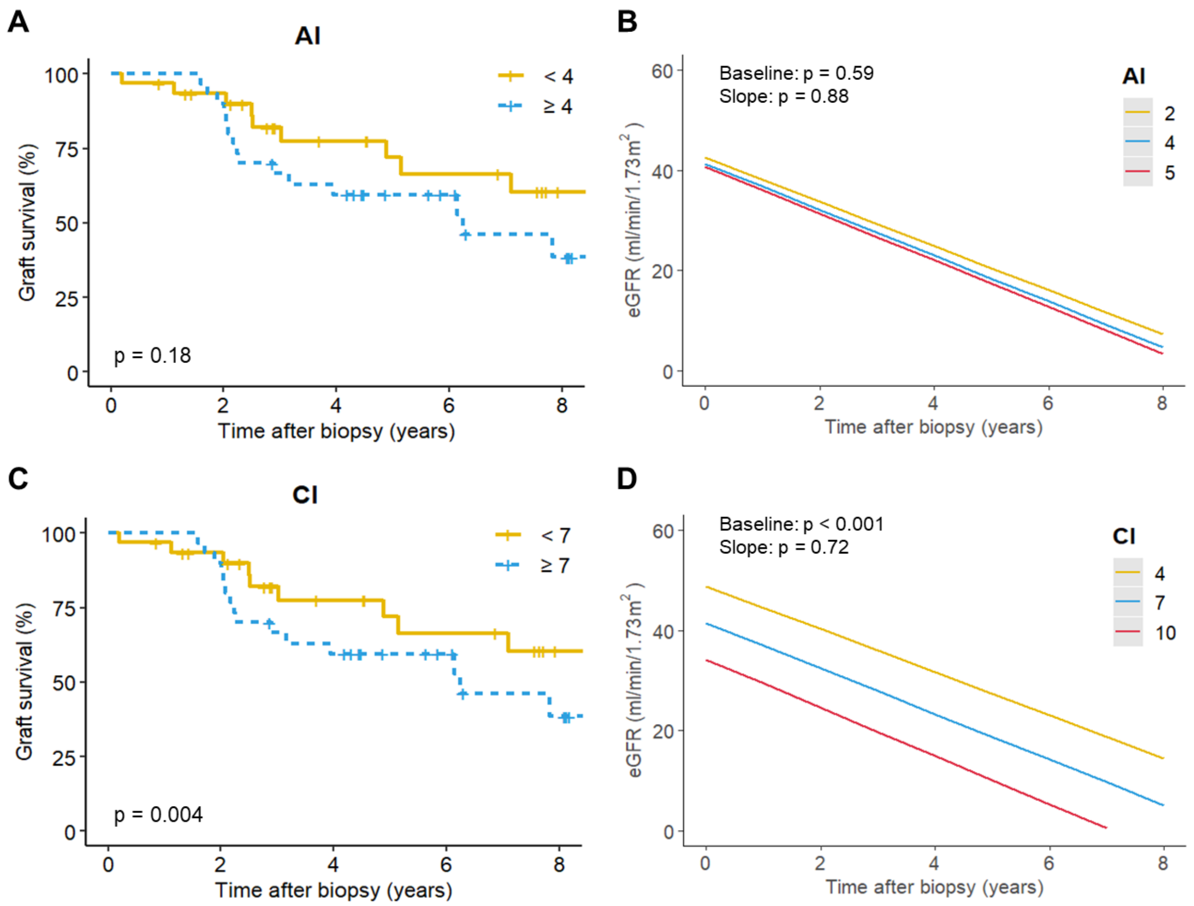

Figure S1. Kaplan Meier curves and predicted estimated glomerular filtration rate (eGFR) slopes for activity index (AI) (A&B) and chronicity index (CI) (C&D). For Kaplan Meier survival analyses, the predictors were dichotomized by their respective medians. Predicted eGFR slopes derived from linear mixed models including an interaction term between the respective predictor and time are shown at the first, second, and third quartile of the respective predictor variable.

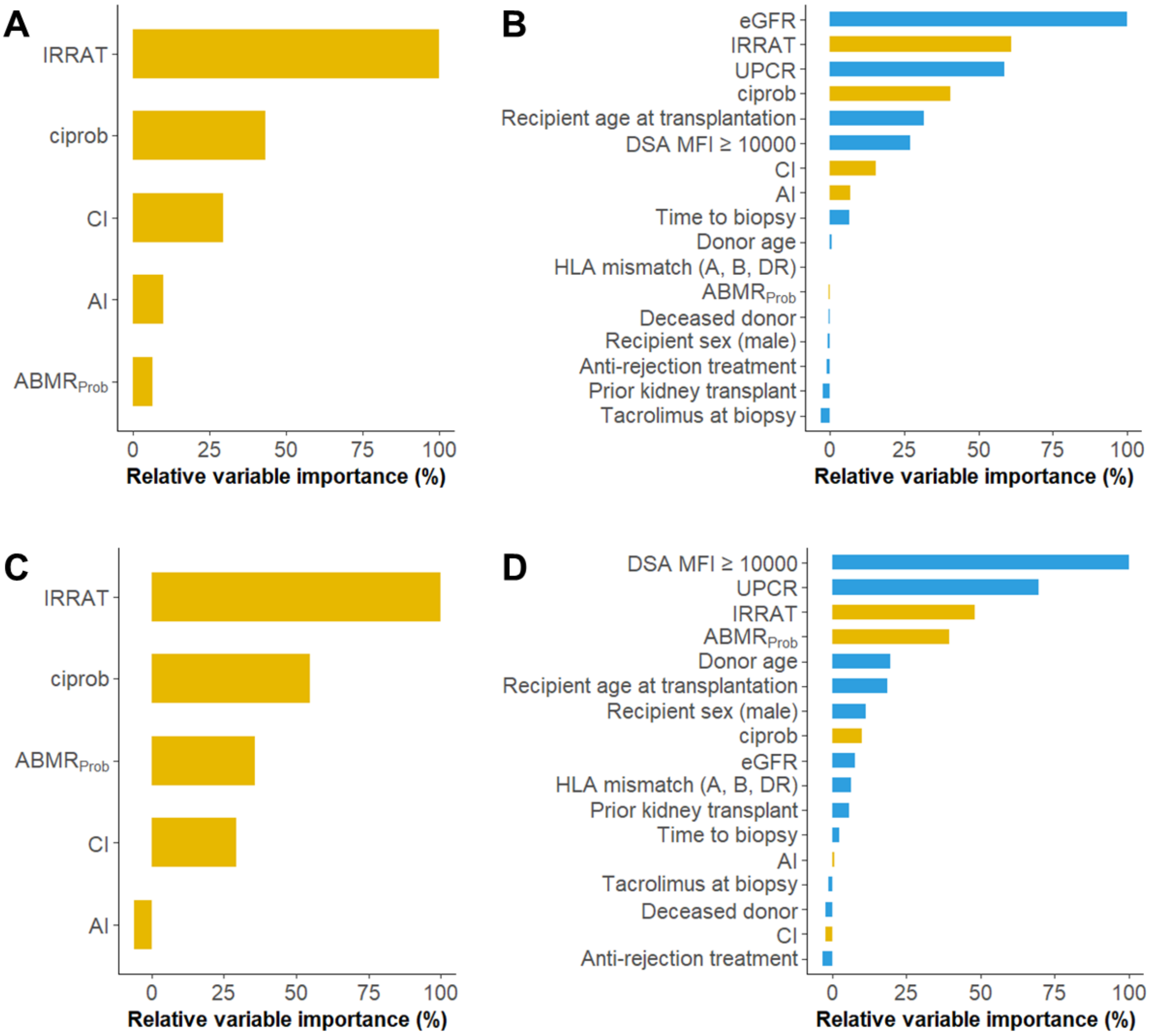

Figure S2. Random forest models to examine the impact of clinical, histologic, and molecular features on death censored graft loss (A, B) and estimated glomerular filtration rate (eGFR) slope (C, D). The prediction models comprised either (A, C) biopsy-related features only (histology and molecular) or a combination of clinical and biopsy-related features (B, D). Individual features were sorted according to their variable importance. Abbreviations: $ABMR_{Prob}$, molecular classifier reflecting the probability of histologic diagnosis of antibody-mediated rejection; AI, activity index (g+ptc+v+C4d); CI, chronicity index (ci+ct+cv+cg[x2]); ciprob, molecular classifier reflecting the probability of histologic ci-lesion score >1; DSA, donor-specific antibody; eGFR, estimated glomerular filtration rate; IRRAT, injury-repair response-associated transcript set; MFI, mean fluorescence intensity; UPCR, urinary protein/creatinine ratio.
